# MELK is an oncogenic kinase essential for metastasis, mitotic progression, and programmed death in lung carcinoma

**DOI:** 10.1038/s41392-020-00288-3

**Published:** 2020-12-02

**Authors:** Qin Tang, Wan Li, Xiangjin Zheng, Liwen Ren, Jinyi Liu, Sha Li, Jinhua Wang, Guanhua Du

**Affiliations:** 1 The State Key Laboratory of Bioactive Substance and Function of Natural Medicines, Beijing, China; 2 Key Laboratory of Drug Target Research and Drug Screen, Institute of Materia Medica, Chinese Academy of Medical Science and Peking Union Medical College, 100050 Beijing, China

**Keywords:** Lung cancer, Oncogenes

## Abstract

Lung cancer is the fastest growth rate of morbidity and mortality in nearly a decade, and remains difficult to treat. Furthermore, the molecular mechanisms underlying its development are still unclear. In this study, bioinformatics analysis showed that MELK was highly expressed in lung cancer and negatively correlated to the survival of lung adenocarcinoma (LUAD). Immunohistochemistry analysis of LUAD patient tissues revealed there were a high level of MELK expression in LUAD. Knockdown of MELK expression inhibits the migration and invasion of LUAD cells, which may be mediated by Twist1, Slug, MMP7, and N-catenin. Overexpression of MELK promoted the growth of LUAD cells in medium, 3D Matrigel, and nude mice. Inhibition of MELK by OTSSP167 arrested cycle of LUAD cells at G2/M phase via PLK1-CDC25C-CDK1 pathway, and triggered apoptosis-mediated pyroptosis. Together, these data indicate that MELK is critical for metastasis, mitotic progression, and programmed death of LUAD and may be a promising therapeutic target for LUAD.

## Introduction

Lung carcinoma is the most cause of incidence and mortality in China and worldwide, and 70% patients were only diagnosted in advanced stage.^1^ Non-small cell lung cancers (NSCLCs), which are divided into lung adenocarcinoma (LUAD), squamous cell lung carcinoma (LUSC) and large cell lung carcinoma (LULC), accounts for 80–85% of lung cancer cases.^2^ The main therapy for NSCLC is molecular targeted agents, such as gefitinib, erlotinib, afatinib, and crizotinib.^3^ However, these agents only function in a small proportion of patients who harbored specific genetic aberrations and mutation, and remain marginally effective in patients without those molecular alterations.^3,4^ Thus, exploring the new therapeutic targets for NSCLC patients is critically needed.

Maternal embryonic leucine zipper kinase (MELK) is a novel oncogene and belongs to an atypical member of the Snfl/AMPK family of serine/threonine kinase.^5^ Numerous studies have demonstrated that MELK is a cell-cycle modulator essential for mitotic progression.^6,7^ Moreover, MELK also participates in other important processes, such as stem cell self-renewal,^8,9^ and apoptosis inhibition.^10,11^ Furthermore, MELK is overexpressed in various cancers and high expression of MELK is related with poor prognosis.^12–15^ However, the roles of MELK in growth, metastasis and death of lung cancer are still unknown.

To explore whether MELK is a novel drug target for lung cancer, we checked the expression of MELK in patients with lung cancer and investigated its roles in the migration, invasion, and growth in lung cancer cells and nude mice. Our results demonstrated that overexpression of MELK promoted growth and metastasis of LUAD cells. In addition, mechanistic analysis revealed that MELK mediated the metastasis of LUAD cells via mediating the expression of Slug, Twist1, MMP7, N-catenin, and E-catenin, regulated the G2/M phase through PLK1-CDC25C-CDK1 pathway, and involved in the inhibition of apoptosis and pyroptosis. MELK may be a promising therapeutic target for LUAD.

## Results

### MELK was overexpressed in LUAD tissues

**Fig. 1:**
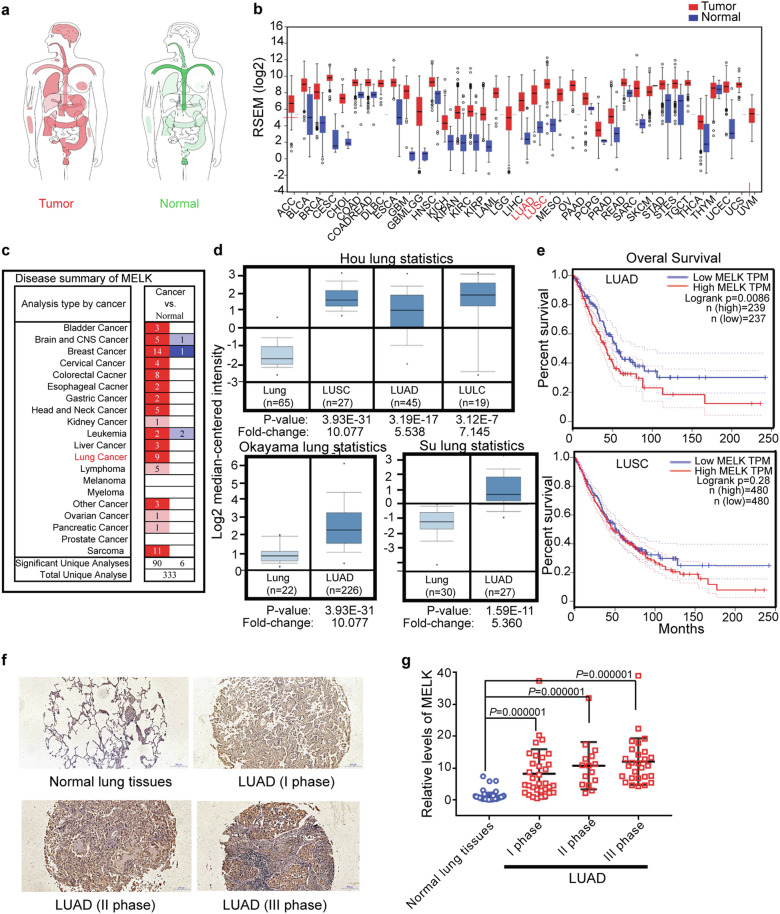
MELK was highly expressed in LUAD tissues. The relationship of MELK expression and lung cancer was investigated by bioinformatics analysis and immunohistochemical analysis of lung cancer tissue chip. **a** MELK expression in tumor and normal tissues was analyzed in website http://gepia.cancer-pku.cn/. **b** MELK expression in tumor and normal tissues was analyzed in website http://gdac.broadinstitute.org/. **c** MELK expression in tumor and normal tissues was analyzed in website https://www.oncomine.org/. **d** MELK expression in lung cancer and normal lung tissues was analyzed in website https://www.oncomine.org/. **e** The survival analysis of LUAD and LUSC patients was performed in website http://gepia.cancer-pku.cn/. **f** Immunohistochemical analysis of MELK expression in LUAD patient tissues chip. The typical photos represented MELK expression in the normal lung tissues adjacent to LUAD and LUAD tissues in various stages. **g** The quantitative analysis of MELK expression in the normal lung tissues adjacent to LUAD and LUAD tissues in various stages. Bars indicates SD, *P* values represented the significant difference between LUAD and normal lung tissues adjacent to LUAD, Student’s *t* test

To explore expression of MELK in lung cancer, bioinformatics analyses were performed using the TCGA, GTEx, and Oncomine databases. Results showed that the expression of MELK was higher in major types of tumors than normal tissues, especially in brain, lung, and breast (Fig. 1a–c). In addition, MELK was highly expressed in three types of lung cancers including LUAD, LUSC, and LULC (Fig. 1d). Survival analysis revealed that the overall survival (OS) of LUAD but not LUSC patients was significantly negatively correlated with the expression of MELK, indicating that MELK could be a potential therapeutic target for LUAD patients (Fig. 1e). Results from TCGA data and immunohistochemistry analysis showed that MELK was remarkably higher expressed in LUAD than normal lung tissues adjacent to LUAD (Fig. 1f, g, Supplementary Fig. S1a), and that expression of MELK was increased with development of LUAD (Fig. 1f, g, Supplementary Fig. S1b).

### MELK promoted migration and invasion of LUAD by upregulating Twsit1, Slug, MMP7, and N-cadherin

**Fig. 2:**
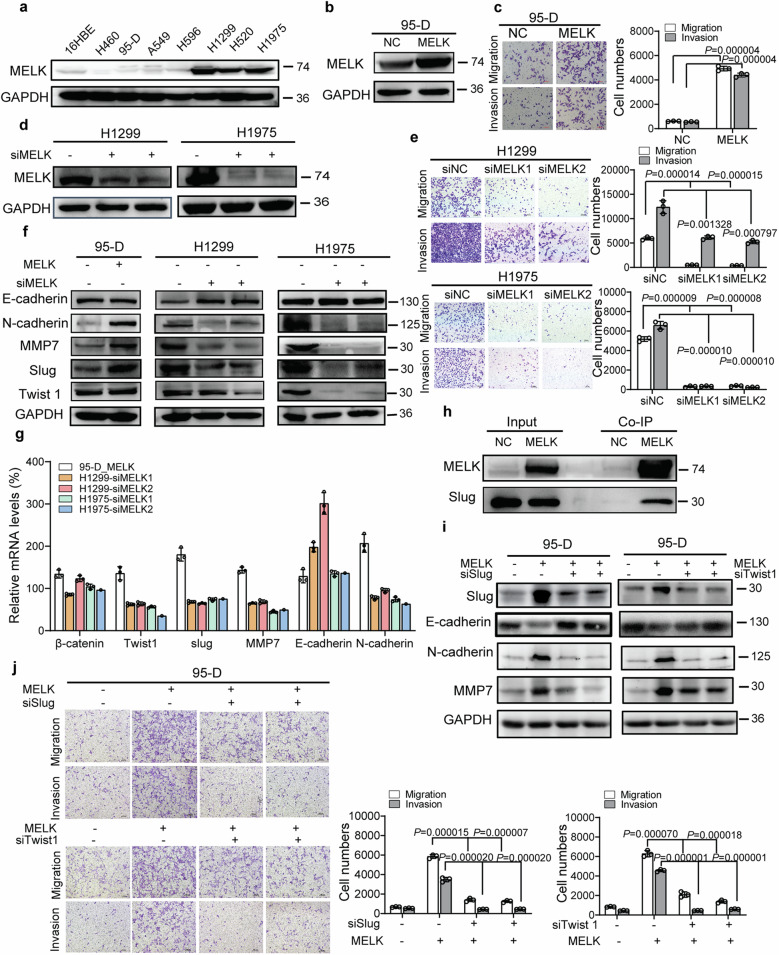
MELK promoted the migration and invasion of LUAD. **a** MELK expression in normal lung cells and lung cancer cells was analyzed by Western blot. **b** LUAD 95-D cells with relative lower levels of MELK were stably transfected with pCMV6-MELK-Myc-DDK (pMELK) for increasing MELK expression and the results were identified by Western blot. **c** The effects of MELK on migration and invasion in 95-D cells were measured by Transwell experiments. Bars indicates SD, *P* values represented the significant difference between 95-D_MELK and 95-D_NC, Student’s *t* test. **d** LUAD H1299 and H1975 cells with relative higher levels of MELK were transfected with siRNA duplexes of MELK for 72 h and detected by Western blot. **e** The effects of MELK on migration and invasion in H1299 and H1975 cells were measured by Transwell experiments. After transfected with siRNA duplex of NC or MELK for 48 h, H1299 and H1975 cells were seeded in the Transwell covered with or without Matrigel for invasion or migration assay. Bars indicates SD, *P* values represented the significant difference between siMELK group and siNC group, Student’s *t* test. **f** The effects of MELK on proteins involved in metastasis were detected by Western blot. H1299 and H1975 cells were transfected with siRNA duplexes of MELK for 72 h and collected for Western blot. **g**. The mRNA levels of those protein which were changed by MELK in protein levels. H1299 and H1975 cells were transfected with siRNA duplexes of MELK for 72 h and collected for qRT-PCR. **h** The interaction of MELK and those protein which were changed by MELK in protein levels. 293T cells were transfected with pNC or pMELK for 24 h and collected for Co-IP analysis. **i** The effects of Slug or Twist1 on MELK-inducing pathway of migration and invasion. 95-D_MELK cells were transfected with siRNA duplexes of Slug for 72 h and analyzed by Western blot. **j** Knockdown of Slug or Twist1 reduced the migration and invasion of 95-D with overexpression of MELK. 95-D_MELK cells were transfected with siRNA duplexes of Slug for 48 h, and the transwell experiments were performed. Bars indicates SD, *P* values represented the significant difference between siSlug or siTwist1 group and corresponding siNC group of 95-D_MELK cells, Student’s *t* test

To investigate the functional roles of MELK in metastasis of LUAD, expression of MELK in LUAD cell lines was checked by Western blot. It was shown in Fig. 2a that there was low MELK expression in 95-D cells whereas there was high MELK expression in H1299 and H1975 cells. Therefore, 95-D cells, H1299, and H1975 cells were used for the following migration and invasion assay. Overexpression of MELK promoted the migration and invasion of 95-D cells (Fig. 2b, c), whereas knockdown of MELK expression inhibited the migration and invasion of H1299 and H1975 cells (Fig. 2d, e). E-cadherin, N-cadherin, MMP2, MMP7, and MMP9 are well-known key regulators in EMT (epithelial–mesenchymal transition) which was closely associated with migration and invasion by degrading and remodeling extracellular matrix. To further explore how MELK promoted the migration and invasion of LUAD, the expression of E-cadherin, N-cadherin, MMP2, MMP7, and MMP9 was checked by Western blot or ELISA. Results showed that MELK obviously changed the expression of E-cadherin, N-cadherin, and MMP7 (Fig. 2f, Supplementary Fig. S2a) but have few effects on expression of MMP2 and MMP9 (Supplementary Fig. S2a). It is well-known that Slug and Twist1 are major transcription factors which mediated EMT. Furthermore, we checked the expression of Slug and Twist1 in 95-D NC and 95-D MELK cells, H1299 NC and H1299 siMELK cells, H1975 NC and H1975 siMELK cells by Western blot. It was shown in Fig. 2f that overexpression of MELK induced expression of Slug and Twist1. On the contrary, knockdown of MELK expression inhibited expression of Slug and Twist1. Finally, to explore how MELK regulates the expression of MMP7, N-cadherin, E-cadherin, Twist1 and Slug, qRT-PCR and Co-IP were performed. The results showed that MELK also altered the mRNA levels of E-cadherin, N-cadherin, MMP7, Slug, and Twist1 (Fig. 2g). The results of Co-IP showed that MELK interacted with and Slug (Fig. 2h).

To further validate mechanisms of MELK in migration and invasion and roles of Twsit1, Slug in MELK mediated migration and invasion, 95-D_MELK cells were transfected with Twist1 siRNA and Slug siRNA for 48 h. Then cell lysis was harvested and protein expression of E-cadherin, N-cadherin, MMP7, Slug, and Twist1 was checked by Western blot and migration and invasion of cells were conducted. These results were shown in Fig. 2i, j. Knockdown of Slug or Twist1 expression in 95-D_MELK cells reduced expression of MMP7, N-cadherin, and increased expression of E-cadherin, and inhibited migration and invasion of cells. In addition, knockdown of MMP7 expression with siRNA duplexes significantly inhibited the migration and invasion of 95-D_MELK and H1299 cells (Supplementary Fig. S2b, c). Taken together, we think that MELK may promote the migration and invasion through upregulating Twsit1, Slug, MMP7, and N-cadherin.

### MELK promoted the growth of LUAD

**Fig. 3:**
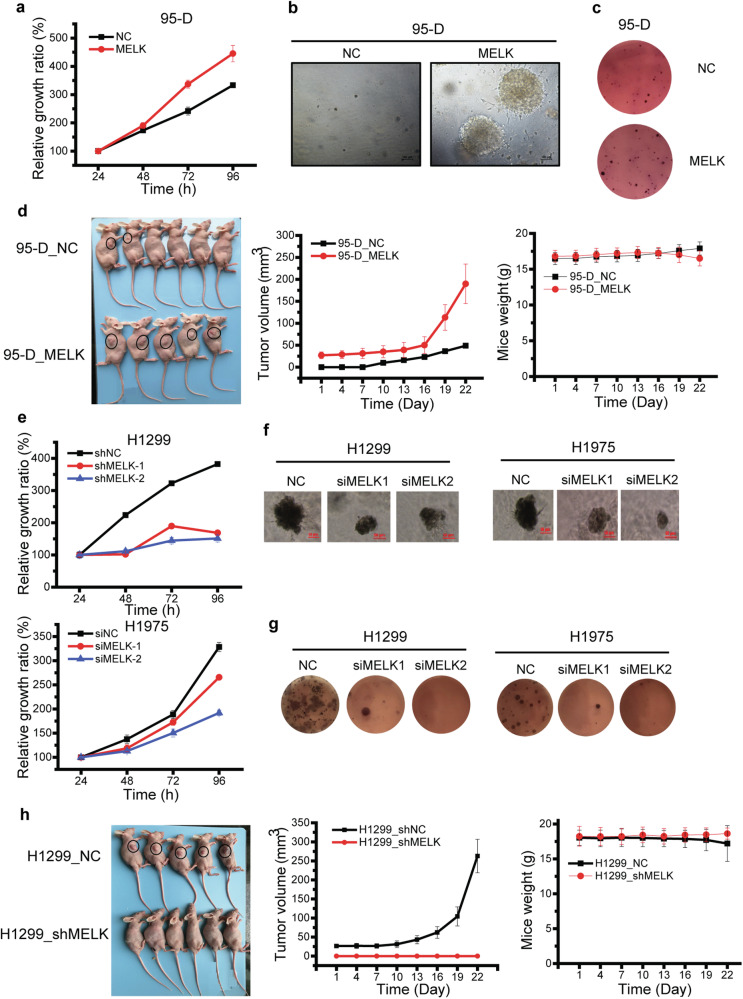
MELK promoted the growth of LUAD. **a** The effect of MELK on the growth of 95-D cells was detected by MTT assay. Totally, 3000 cells/well of 95-D_NC or 95-D_MELK were seeded in 96-well, and the cell numbers were measured by MTT assay after incubation for 24, 48, 72, and 96 h. The relative growth rate were calculated with formula: relative growth rate (%) = (OD_570-t_)/(OD_570–24 h_) × 100. OD_570-t_ represented the value of absorbance at 570 nm at indicated time while OD_570–24 h_ represented the value of absorbance at 570 nm at 24 h. **b** The effect of MELK on the growth of 95-D cells in 3D Matrigel. Totally, 1000 cells/well of 95-D_NC or 95-D_MELK were seeded in 96-well and cultured in DMEM medium with 10% FBS, 50% Matrigel, streptomycin (100 μg/mL) and penicillin (100 U/mL) for 2 weeks. **c** The effect of MELK on the growth of 95-D cells in soft agar. Totally, 3000 cells/well of 95-D_NC or 95-D_MELK were seeded in 6-well and cultured in 0.4% agar for 2 weeks. **d** The effect of MELK on the growth of 95-D cells in nude mice. A total of 5 × 10^6^ 95-D_NC or 95-D_MELK cells were seeded in the right frank of nude mice and cultured for 1 month. **e** The effect of MELK on the growth of H1299 and H1975 cells were detected by MTT assay. Totally, 3000 cells/well of H1299-NC or H1299-shMELK-1/2 were seeded in 96-well, and the cell numbers were measured by MTT assay after incubation for 24, 48, 72, and 96 h. Totally, 3000 cells/well of H1975 were transfected with siRNA duplexes of NC or MELK and then measured by MTT assay. **f** The effect of MELK on the growth of H1299 and H1975 cells in 3D Matrigel. After transfected with siRNA duplexes of NC or MELK for 48 h, cells were mixed with Matrigel at the ratio of 1:1, seeded into 96-well at the density of 1000 cells/well and cultured for 4 weeks. **g** The effect of MELK on the growth of H1299 and H1975 cells in soft agar. After transfected with siRNA duplexes of NC or MELK for 48 h, cells were seeded in 6-well at the density of 3000 cells/well and cultured in 0.4% agar for 2 weeks. **h** The effect of MELK on the growth of H1299 cells in nude mice. Totally, 5 × 10^6^ H1299-shNC or H1299-shMELK cells were seeded in the right frank of nude mice and cultured for 1 month

To explore the effects of MELK on the growth of LUAD, the experiments of proliferation, growth in 3D Matrigel, colony formation, and nude mice transplantation were carried out. The results showed that overexpression of MELK increased the proliferation of 95-D (Fig. 3a) and promoted the growth of 95-D cells in 3D Matrigel (Fig. 3b), soft agar (Fig. 3c), and nude mice (Fig. 3d). In contrast, the knockdown of MELK expression in H1299 and H1975 significantly inhibited the proliferation (Fig. 3e) and growth in 3D Matrigel (Fig. 3f) and soft agar (Fig. 3g). Moreover, the knockdown of MELK expression in H1299 also reduced the tumor growth in nude mice (Fig. 3h). Immunohistochemistry analysis of tumors with Ki-67 antibody was shown in Supplementary Fig. S3, which showed no significant difference of Ki67 expression between 95-D NC and 95-D_MELK cells. Altogether, MELK plays a vital role in the growth of LUAD.

### MELK regulated the cell cycle progression of LUAD via PLK1-CDC25C-CDK1 pathway

**Fig. 4:**
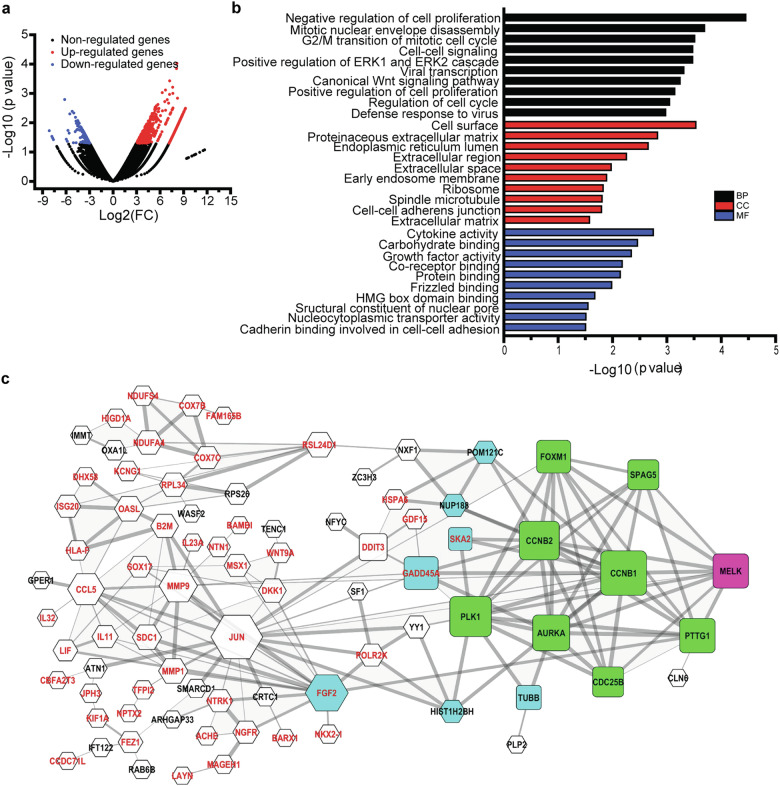
MELK played vital roles in cell cycle progress of LUAD. H1299_NC, H1299_shMELK1, and H1299_shMELK2 (passage 4) were harvested in TRizol for RNA isolation and sequencing. **a** The differentially expressed genes in H1299_shMELK1 and H1299_shMELK2 groups. **b** The GO enrichment analyses of the differentially expressed genes. **c** The protein interaction analyzed on STRING website. Black and red words, respectively represented the genes which are downregulated and upregulated by knockdown of MELK. Squre and hexagon separately indicated the genes associated to cell cycle and other genes. Pink, green, and blue colors referred to MELK genes, genes interacted with MELK and their interacting genes, respectively. The thickness of line between two genes indicated the strength of interaction

**Fig. 5:**
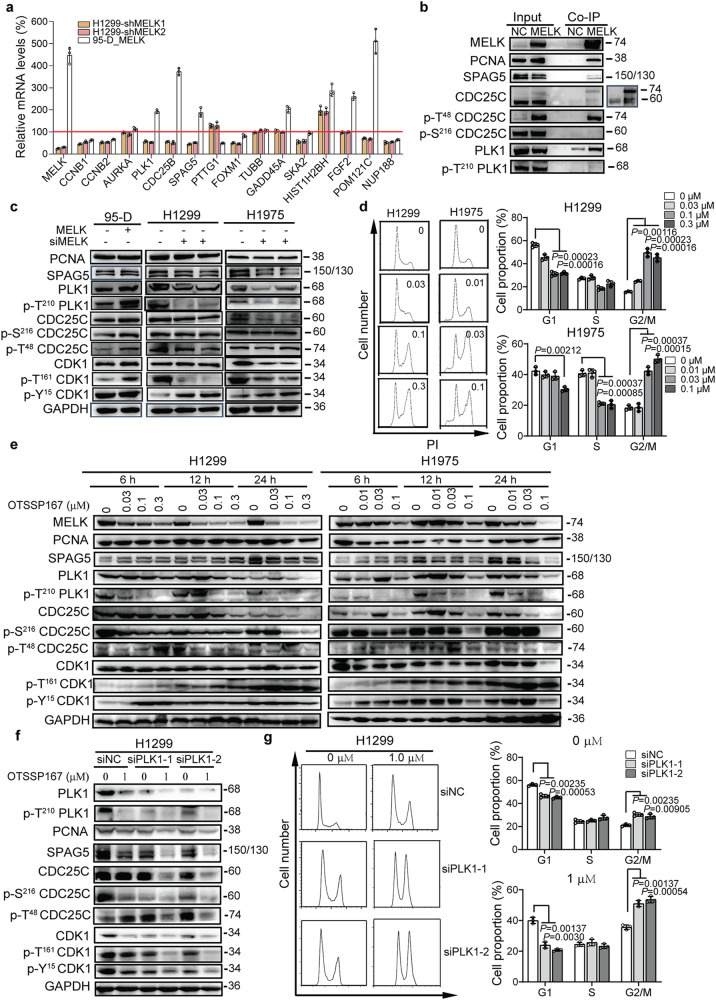
MELK regulated the cell cycle of LUAD cells via the PLK1-CDC25C-CDK1 pathway. **a** The mRNA levels of genes related to MELK. The red line represented the value of relative mRNA level was 100%. **b** The protein in G2/M phase regulation interacted with MELK. 293T cells were transfected with pNC or pMELK for 24 h and collected for Co-IP analysis. **c** The expression of proteins in G2/M phase regulation was regulated by MELK. **d** OTSSP167 arrested cell cycle of H1299 and H1975. Cell cycle was arrested at G2/M phase by OTSSP167. Cells were treated with OTSSP167 at the indicated concentration for 48 h, and analyzed by PI staining. Bars indicates SD, *P* values represented the significant difference between OTSSP167 treated group and negative control in H1299 and H1975, Student’s *t* test. **e** The effects of MELK on proteins related to G2/M phase regulation. Cells were treated with OTSSP167 at the indicated concentration for 6, 12, and 24 h, then detected by Western blot. **f** Knockingdown of PLK1 expression enhanced the effects of OTSSP167 on regulators in G2/M phase. H1299 cells for transfected with siNC or siplk1-1/2 for 48 h and treated with OTSSP167 for 24 h, followed by Western blot assay. **g** Decreasing the levels of PLK1 contributed to arresting cell cycle of H1299 at G2/M induced by OTSSP167. H1299 cells were transfected with siNC or siplk1-1/2 for 48 h and treated with OTSSP167 for 24 h, followed by PI staining. Bars indicates SD, *P* values represented the significant difference between siplk1 group and corresponding siNC group, Student’s *t* test

To explore the molecular mechanism of MELK in LUAD, RNA-seq analysis was performed. Stable cell lines H1299_NC, H1299_shMELK1, and H1299_shMELK2 (passage 4) were harvested and sequenced. Compared with H1299_NC group, 463 genes were upregulated and 178 genes were downregulated in H1299_shMELK1 and H1299_shMELK2 groups (Fig. 4a). The top ten enrichment ontologies of these genes using the DAVID website were listed in Fig. 4b. The results showed that MELK may involve in G2/M phase regulation. The constructed protein–protein interaction network by Cytoscape indicated MELK were interacted with SPAG5, PTTG1, CCNB1, CCNB2, FOXM1, AURKA, CDC25B, and PLK1, whose expression levels were all downregulated by the knockdown of MELK in the RNA-seq data (Fig. 4c). Furthermore, qRT-PCR validated that only PLK1, CDC25B, and SPAG5 were decreased in H1299_shMELK1 and H1299_shMELK2 groups and increased in 95-D_MELK group (Fig. 5a).

To investigate downstream targets of MELK in G2/M phase, Co-IP experiment was performed. Results showed that MELK interacted with PCNA, SPAG5, CDC25C, p-T^48^ CDC25C, and PLK1 but not with p-S^216^ CDC25C, p-T^210^ PLK1, and CDK1 (Fig. 5b, the data for CDK1 was not shown). Results also showed that MELK promoted the phosphorylation of PLK1 at T^210^, CDC25C at T^48^ and CDK1 at T^161^ and knockdown of MELK led to the phosphorylation of CDK1 at Y^15^ (Fig. 5c). In addition, the specific inhibitor of MELK, OTSSP167, arrested cell cycle of H1299 and H1975 at G2/M phase (Fig. 5d), and reduced the levels of SPAG5, PLK1, p-T^210^ PLK1, CDC25C, and p-S^216^- CDC25C in a concentration- and time-dependent manner (Fig. 5e). Knockdown of PLK1 with siRNA duplex enhanced the effects of OTSSP167 in regulators (Fig. 5f) and arrest of G2/M (Fig. 5g). Moreover, antibody of PLK1 could pull down MELK (Supplementary Fig. S4), further suggesting that MELK interacted with PLK1. In summary, MELK regulated the cell cycle through PLK1-CDC25C-CDK1 pathway.

### Inhibition of MELK induced the apoptosis-mediated pyroptosis of LUAD

**Fig. 6:**
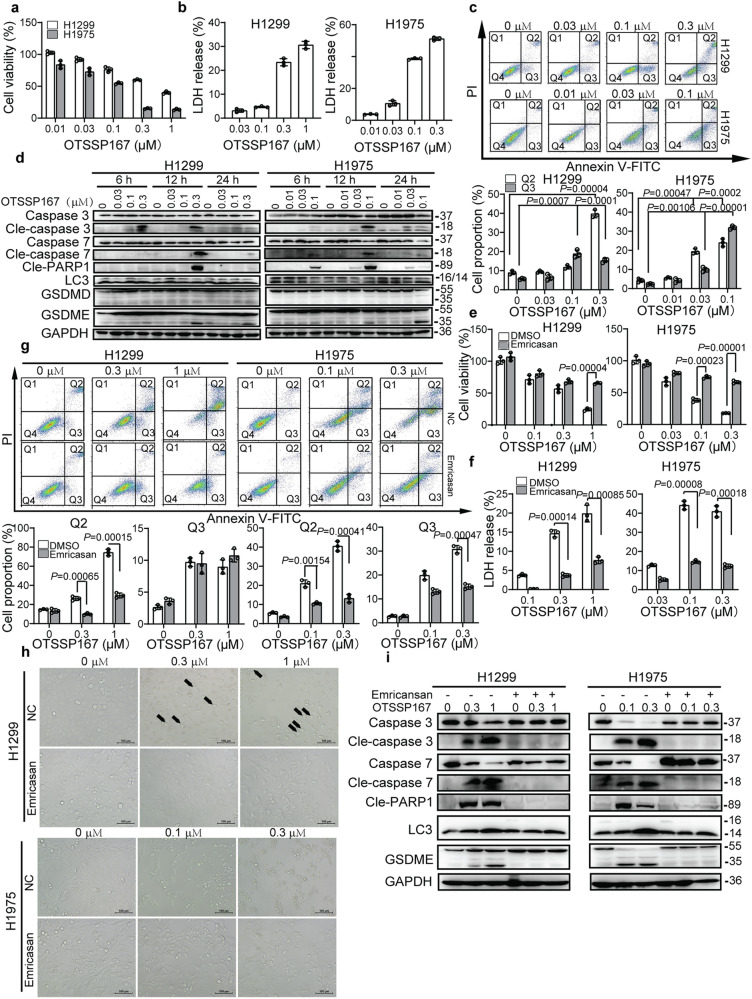
MELK inhibitor induced apoptosis-mediated pyroptosis in LUAD. **a**. OTSSP167 inhibited the cell viability of H1299 and H1975 cells. Cells were treated with OTSSP167 for 24 h and detected by MTT assay. **b** OTSSP167 triggered the LDH release of H1299 and H1975 cells. Cells were treated with OTSSP167 for 24 h and detected by LDH assay. **c** OTSSP167 increased the apoptosis rate of H1299 and H1975 cells. Cells were treated with OTSSP167 for 24 h, followed by apoptotic assay. The Q1–Q4 quadrants represented dead, late-apoptotic, early-apoptotic, and normal cells, respectively. Bars indicates SD, *P* values indicated significant differences between the OTSSP167-treated group and control group, Student’s *t* test. **d** The alteration of proteins related to programmed death was caused by OTSSP167. Cells were treated with OTSSP167 for 6, 12, and 24 h, followed by Western blot. **e** Emricasan eliminated the influence of OTSSP167 on cell viability of lung cancer. Cells were pretreated with 50 μM Emricasan for 24 h and treated with OTSSP167 for another 24 h. Bars indicates SD, *P* values indicated significant differences between the Emricasan-treated group and non-Emricasan-treated group, Student’s *t* test. **f** The effects of OTSSP167 on LDH release of lung cancer were abolished by Emricasan. Bars indicates SD, *P* values indicated significant differences between the Emricasan-treated group and non-Emricasan-treated group, Student’s *t* test. **g** Emricasan decreased the apoptotic cells increased by OTSSP167. Bars indicates SD, *P* values indicated significant differences between the Emricasan-treated group and non-Emricasan-treated group, Student’s *t* test. **h** The morphological alteration induced by OTSSP167 was restored by Emricasan. **i** The effects of OTSSP167 on proteins related with programmed death were reversed by Emricasan

To explore the mechanism by which inhibiting MELK mediated the death of LUAD, the specific inhibitor of MELK, OTSSP167, was used to treat cells. As showed in Fig. 6a, OTSSP167 inhibited the growth of H1299 and H1975 cells in a concentration-dependent manner. Meanwhile, OTSSP167 of high concentration also triggered the release of LDH in H1299 and H1975 cells (Fig. 6b), indicating that OTSSP167 may induce pyroptosis. Apoptotic assay showed that OTSSP167 induced apoptosis of cells (Fig. 6c). Furthermore, results of Western blot showed that OTSSP167 increased the levels of cleaved caspase 3/7 and PARP1 from 6 h treatment, then their levels peaked at 12 h and decreased at 24 h (Fig. 6d). After treatment of OTSSP167 for 12 h, the N-terminus of GSDME rather than GSDMD (35 kDa) which participated in pyroptosis process were obviously detected in both H1299 and H1975 cells. It is well known that Caspase-1 is a key marker and regulator of pyroptosis. It was shown in Supplementary Fig. S5a that expression of Caspase-1 was also increased by OTSSP167 from 6 h. Furthermore, knockdown of GSDME expression with siRNA duplex significantly restored the cell viability of H1299 cells (Supplementary Fig. S5b). Thus, the result suggested that OTSSP167 might induce the GSDME-mediated pyroptosis. Otherwise, OTSSP167 affected little on the levels on LC3, the marker of autophagy. Furthermore, the effects of OTSSP167 on cell viability, LDH release, apoptosis, morphology and related proteins were almost eliminated by Emricasan, a pan-inhibitor of caspase (Fig. 6e–i), suggesting that inhibition of MELK may induced the apoptosis-mediated pyroptosis of lung cancer.

## Discussion

Lacking essential molecular targets is the vital cause for the high mortality of patients with lung carcinoma. In this study, both the bioinformatics and immunohistochemistry analyses showed that MELK was upregulated in LUAD tissues (Fig. 1). Notably, the overexpression of MELK promoted the migration and invasion of lung cancer cells (Fig. 2). Remarkably, MELK is required for survival and proliferation of LUAD in vitro and in vivo (Fig. 3). More interestingly, inhibiting the kinase activity of MELK arrested the LUAD cells at G2/M phase and induced robust apoptosis as well as pyroptosis (Figs. 5d and 6a–d). Thus, MELK is potentially a novel oncogenic driver of LUAD and a promising target for small molecule-based therapeutic intervention.

Construction of MELK regulation network will guide the design and application of MELK inhibitors. Proliferation and metastasis constitute the two key elements of malignancy of lung carcinoma. Tumor metastasis is a complex process where epithelia–mesenchymal transition (EMT) is the initial step.^16,17^ When EMT occurs, the cells will lose the expression of E-cadherin, acquire the expression of N-cadherin and increase the expression of metalloproteinases (MMPs).^18,19^ The process is controlled by EMT transcription factors, such as the SNAIL family zinc finger transcription factors (SNAIL1 and SNAIL2), the TWIST family basic helix–loop–helix transcription factors (TWIST1 and TWIST2), and the zinc finger E-box binding homeobox proteins (ZEB1 and ZEB2).^20–22^ Previous studies on MELK concentrated more on its function in survival rather than metastasis. Here, we firstly reported that MELK may interact with Slug, upregulate the expression of MMP7 and N-cadherin, as well as decrease the expression of E-cadherin through Slug or Twist1 to promote the migration and invasion of LUAD (Fig. 2e–j).

Likewise Cyclin B, the expression of MELK is cyclical and maximal at mitotic (M) phase, so M-phase is the pivotal time for MELK regulation.^23^ Molecularly, MELK associates with and phosphorylates S^323^ of CDC25B and involves in the inhibition of spliceosome assembly.^24,25^ Joshi et al.^26^ report that MELK drive the activation of FOXM1 to increase the expression of mitotic regulatory genes in GSCs. Wang et al.^27^ identify that MELK directly interacts with eukaryotic translation initiation factor 4B (eIF4B) to phosphorylate it at S^406^ for protein synthesis at M-phase. Except those, whether other mitotic regulators are controlled by MELK is still unknown. The master mitotic driver is Cyclin B-CDK1 complex which is completely activated depending on the de-phosphorylation of CDK1 at Y^15^/T^14^ and phosphorylation at T^161^.^28^ The phosphorylated levels of CDK1 at Y^15^/T^14^ are controlled by positive regulators Wee l and Myt as well as negative relators CDC25 family.^29,30^ Gheghiani et al pointed out that Polo-like kinase 1 (Plk1) is rapidly activated shortly before CyclinB1-Cdk1 during entry into mitosis to associates with the Cdc25C1 phosphatase and induces its phosphorylation.^31^ To explore mechanism of MELK in LUAD, RNA-seq analysis, and co-IP were carried out and results showed that MELK interacted with SPAG5, PCNA, CDC25C at M-phase (74-kDa) and PLK1. Knockdown MELK expression by siRNA duplex and inhibiting its function by OTSSP167 reduced the expression of SPAG5, T^210^ phsphorylation of PLK1, S^210^ phsphorylation of CDC25C and increased Y^15^ phsphorylation of CDK1 (Fig. 5c, e). Furthermore, knockdown of PLK1 with siRNA duplex enhanced the effects of OTSSP167 in regulators (Fig. 5f) and arrested cell cycle at of G2/M stage (Fig. 5g). Taken together, MELK may modulate G2/M phase via PLK1-CDC25C-CDK1 pathway.

Lin et al.^10^ imply that MELK specifically interacts with BcL-G_L_ to trigger the apoptosis. However, when the H1299 and H1975 cells were treated with the specific inhibitor of MELK, OTSSP167, the altered morphologies of these cells were different from apoptotic cells. OTSSP167 promoted the swelling of H1299 cells characterized by large bubbles from the plasma membrane (the typical pyrototic morphology) and elongated intercellular bridges of H1975 cells, indicating that OTSSP167 may induce other type of death (Fig. 6h). Furthermore, we found that OTSSP167 increased the LDH release and apoptotic cells in a dose-dependent manner (Fig. 6b, c). As showed in Fig. 6d, OTSSP167 cleaved the caspase 3/7 (executors of apoptosis) and activated GSDME rather than GSDMD (markers of pyroptosis^32,33^). However, it affected little on the transition from I to II type of LC3 (marker of autophagy). These results suggested that apoptosis and pyroptosis may be the main processes of death induced by the inhibition of MELK. Moreover, the apoptosis and pyroptosis induced by OTSSP167 were alleviated by Emricansan (Fig. 6e–i), a pan-inhibitor of caspase, suggesting that OTSSP167 could trigger the pytotosis via the apoptotic pathway.

**Fig. 7:**
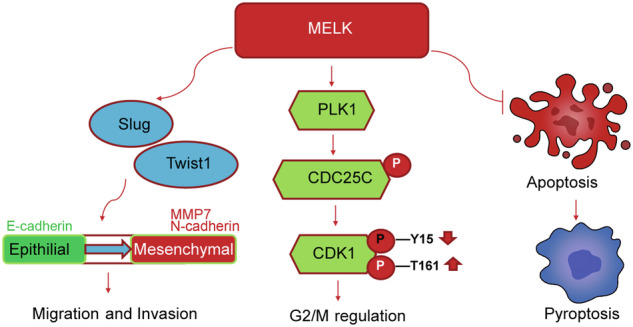
Proposed mechanistic scheme: MELK is critical for metastasis, mitotic progression, and programmed death of LUAD. MELK promoted EMT by regulating expression of Slug and Twist1. In addition, MELK promoted cell cycle by regulating PLK1-CDC25C-CDK1 pathway. In contrast, Inhibiting MELK arrested cell cycle and induced apoptosis-mediated pyroptosis

Overall, we elucidated the functional roles and mechanism of MELK in LUAD. The overexpression of MELK promoted the growth of LUAD by regulating the PLK1-CDC25C-CDK1 pathway, and increased the migration and invasion of LUAD through EMT-transcription factors mediated degradation of MMP7, and upregulation of N-cadherin. The inhibition of MELK arrested the LUAD at the G2/M phase and triggered pyroptosis via apoptosis (Fig. 7). These findings provide a rational foundation for designing MELK inhibitors. MELK may be a promising therapeutic target for LUAD.

## Materials and methods

### Cell culture

The human lung cancer cell lines 95-D, NCI-H460, A549, NCI-H596, NCI-H1299, NCI-H520, NCI-H1975, and human bronchial epithelial cell line 16-HBE were purchased from the GuangZhou Jennio Biotech Co., Ltd. (Guangdong, China). Human renal epithelial cells 293T were preserved in the laboratory. Cells were maintained with DMEM supplemented with 10% FBS, streptomycin (100 μg/mL), and penicillin (100 U/mL). The cells were cultured at 37 °C in an incubator with a humidified atmosphere of 5% CO_2_.

### Chemical agents and antibodies

3-(4,5-dimethyl-2-thiazolyl)-2,5-diphenyl-2-H-tetrazolium bromide (MTT), PI dyes, RNase, penicillin, and streptomycin were bought from Sigma-Aldrich (St. Louis, MO, USA). Emricasan, OTSSP167, and Z-DEVD-FMK were purchased from Targetmol (Shanghai, China). Fetal bovine serum (FBS) and Dulbecco’s Modified Eagle Medium (DMEM) were bought from Gibco (Grand Island, NY, USA). The goat-anti-rabbit secondary antibody, rabbit monoclonal N-cadherin, E-cadherin, Vimentin, Slug, Beta-catenin, PCNA, SPAG5, CDC25C, phospho-T^48^ CDC25C, phospho-S^216^ CDC25C, PLK1, phospho-T^210^ PLK1, CDK1, phospho-T^161^ CDK1, phospho-Y^48^ CDK1, caspase 3, cleaved caspase 3 (cle-caspase 3), and cleaved PARP1 (cle-PARP1) were obtained from Cell Signaling Technology (Danvers, MA,USA). Mouse monoclonal GAPDH, rabbit monoclonal Twist1 and rabbit polyclonal caspase 1 were purchased from Proteintech (Wuhan, China). Rabbit monoclonal GSDMD and GSDME, rabbit polyclonal Ki-67 were purchased from Abcam (Cambridge, MA, USA). The details for ID code and dilution of antibodies were listed in the supplemental materials (Supplementary Table S1).

### Bioinformatics analyses

The mRNA level of MELK in tumor versus normal tissues was analyzed in website http://gepia.cancer-pku.cn/, https://www.oncomine.org/, and http://gdac.broadinstitute.org/. The mRNA level of MELK in lung cancer was analyzed in website https://www.oncomine.org/. The survival analysis of MELK in lung cancer was determined in website http://gepia.cancer-pku.cn/. The expression of MELK in LUAD was analyzed according to database of The Cancer Genome ALTAS (TCGA).

### Immunohistochemistry analysis on tissue chip and xenograft tumors

The chip of LUAD (Cat No. HLugA150CS03) was purchased from SuperChip in Shanghai, China. The chip, respectively, contained 75 tumor and adjacent tumor tissues, and included I (33 cases), II (15 cases), III (25 cases), and IV (2 cases) phase LUAD tumor tissues. After routinely dewaxing and hydration, antigen in specimens proceeded to repaired by microwaving in citric saline at 95 °C for 90 s. Then, the endogenous enzymes in specimens were removed by 3% hydrogen peroxide. After permeated by 0.1% Trinton X-100 and blocked with 5% bovine serum albumin, the chip was incubated with primary antibody against mouse anti-MELK antibody (1:200) at 4 °C overnight. The chip was balanced at room temperature for 30 min, washed by PBS and incubated with horseradish peroxidase-conjugated goat anti-mouse IgG (DAKO, Wuhan, China) for 60 min at room temperature. MELK expressions were visualized by DAB (DAKO, Wuhan, China) staining. The chip was fully scanned by Pannoramic Scan (3DHISTECH Ltd. Hungary) and quantitatively analyzed by Image-Pro Plus 6.0 software. Immunohistochemistry analysis of xenograft tumors with Ki-67 antibody was also carried out.

### Migration and invasion assay

This experiment was performed using 6.5 mm Transwell^®^ with 8.0 µm Pore Polycarbonate Membrane Insert (Corning, NY, USA). For migration assay, 2 × 10^4^ cells were seeded into the upper chamber and cultured at 37 °C for 3–4 h. The medium was replaced by fresh medium without serum, and medium contained 10% FBS were added to the bottom chamber. After 19 h, the migrated cells were stained by Crystal Violet 0.1% (m/v) and the total number of cells was counted with fluorescence microscopy (Nikon Eclipse Ti-U, Japan). For invasion assay, the upper chamber was coated with Matrigel^TM^ which was diluted by medium without serum at the ratio of 1:7. Totally, 1 × 10^5^ cells were seeded into the upper chamber and cultured at 37 °C for 22 h, followed by stained by Crystal Violet. To identify the role of Slug and Twist1 in migration and invasion induced by MELK, 95-D_MELK cells were transfected with siRNA duplexes of Slug, Twist1, or NC for 48 h, followed by the above procedures.

### Transfection and stable-cell line construction

siRNA duplexes were obtained from Genepharm (Shanghai, China) and transfected into H1299 or H1975 cells using Lipofectamine 3000 according to manufacturer’s instructions. The sequences of two siRNA or shRNAs targeting MELK gene were listed as follow: 5′-GCATTCTGCTTCTTCAACA-3′ and 5′-CCAAAGACUCCAGUUAAUA-3′. For stable MELK over-expressed 95-D cell line construction, pCMV6-MELK-Myc-DDK (pMELK) and pCMV6-Myc-DDK (pNC) were purchased from ORIGENE (Beijing, China) and transfected into 95-D cells for 24 h, followed by being seeded into 10 cm dish with 1000 cells/dish. After cultured for 24 h, 1.2 mg/ml G418 were added for screening. The culture medium was replaced with fresh medium containing 10% FBS and 1.2 mg/ml G418. The stable cell clones which had high MELK expression were selected and cultured with DMEM medium containing 10% FBS and 1.2 mg/ml G418. For stable MELK knockingdown H1299 cell line construction, two shRNAs (target sequences): 5′-GCATTCTGCTTCTTCAACA-3′ and 5′-CCAAAGACTCCAGTTAATA-3′, were cloned into the pGPH1/GFP/Neo. The concentration of G418 used in screening of stable MELK knocking-down H1299 cells was 0.6 mg/ml. To identify the role of Slug, Twist1 and PLK1 in MELK-mediated pathway, the siRNA duplex of Slug, Twist1 and PLK1 were used. The sequences were listed in the supplemental materials section (Supplementary Table S2).

### RNA extraction, cDNA synthesis, and qRT-PCR

After indicated treatments, cells were harvested in Trizol. After mixing with 1/5 volume of chloroform, the mixture was centrifuged at 12,000 rpm for 15 min and supernatants were transferred into new, clear centrifuge tubes. An equal volume of isopropanol was added into each supernatant and gently mixed. After incubation at room temperature for 30 min, the mixture was centrifuged at 12,000 rpm for 15 min. The pellets were washed once with 75% ethanol and dissolved in RNase-free water at an appropriate volume. After quantification of RNA, cDNA was synthesized using PrimeScript^TM^ RT 1st Master Mix (Takara, Japan) according to the manufacturer’s instructions. Quantitative real-time RT-PCR (qRT-PCR) was performed using TB Green® Premix Ex Taq^TM^ II (Tli RNaseH Plus) (Takara, Japan). The primers are listed in the Supplemental Materials section (Supplementary Table S3). GAPDH was served as internal control.

### Western blot

After the indicated treatments, cells were harvested and resuspended in RIPA buffer for protein extraction. Protein concentration was determined using a BCA assay kit from APPLYGEN (Beijing, China). Aliquots of 80–100 μg of protein were separated by 10% sodium dodecyl sulfate polyacrylamide gel electrophoresis, and then transferred onto PVDF membranes (Merck Millipore Ltd., Germany). The membranes were blocked with TBST containing 5% nonfat milk at room temperature for 1 h and incubated with the indicated antibodies at 4 °C overnight. Subsequently, the membranes were washed three times with TBST, and incubated with secondary antibody conjugated to horseradish peroxidase at room temperature for 1 h. Finally, the membranes were washed three times with TBST and incubated with ECL reagents. The membranes were examined using a chemiluminescence photodocumentation system (Tanon, Beijing, China) photographed and quantitated.

### Co-immunoprecipitation (Co-IP)

293T cells were seeded into 100 mm dish and cultured for 24 h to a 70–80% confluence. Totally, 1 μg pMELK and pNC were transfected into 293T cells, separately. After cultured for 24 h, the cells were collected. After dissolved in 550 μL lysis buffer on ice for 30 min and the mixture was centrifuged at 12,000 rpm for 30 min. Totally, 40 μL supernatant was mixed with 10 μL 5× loading buffer and denaturalized at 100 °C for 10 min, while the others were mixed with 10 μL DDK-conjugated agarose beads and gently shook overnight at 4 °C. The mixture was centrifuged at 3000 rpm for 3 min and the pellets were washed by TBS for five times. Finally, the pellets were resuspended in 30 μL 5× loading buffer, denaturalized at 100 °C for 10 min and detected by Western blot.

### MTT assay

After indicated treatments, cells were washed once with PBS and incubated with serum-free DMEM containing 0.5 mg/mL MTT for 3–4 h. The supernatants was carefully removed and discarded, and the formazan was dissolved in 100 μL dimethyl sulfoxide, followed by measurement with a SpectraMax M5 plate reader (Molecular Devices, Shanghai, China) at 570 nm. For growth assay, cells were seeded into 96-well plates at 3 × 10^3^ per well and cultured for 24, 48, 72, and 96 h, respectively. For drug treatment, cells were cultured for 24 h and administrated with or without Emricasan for another 24 h, followed by OTSSP167 treatment for 24 h.

### 3D Matrigel

Matrigel^TM^ was purchased from BD Bioscience (Franklin Lakes, NJ, USA). Cells (1000/well) were mixed with the same volume of Matrigel^TM^ on the ice and seeded into 96-well plate. After solidified at 37 °C for 30 min, the medium was added in wells. After cultured for 2–3 weeks, the colonies were visualized by fluorescence microscopy (Nikon Eclipse Ti-U, Japan).

### Colony formation assay

Low melting point agarose was bought from Thermo Fisher Scientific (Waltham, MA, USA). The assays were performed in a 6-well plate. Cells were suspended in medium containing 0.4% agar and plated onto a layer of 0.7% agar (5000 cells/well in 1.5 ml medium, 2 ml bottom agar). After cultured for 2–3 weeks, the colonies were stained with MTT.

### Xenograft assay in nude mice

Animal studies were approved by The Committee of Animal Care & Welfare at the Institute of Materia Medica, Chinese Academy of Medical Science & Peking Union Medical College (No. 0005265). About five weeks old athymic nude mice (16–18 g) were purchased by the Animal House in the Department of Animal Care Center at Institute of Materia Medica, Chinese Academy of Medical Science & Peking Union Medical College. The animals were housed at 24 °C with ad libitum access to food and water. All experimental procedures were carried out in accordance with institutional guidelines for the care and use of laboratory animals at the Institute of Materia Medica, Chinese Academy of Medical Science & Peking Union Medical College and the National Institutes of Health Guide for Care and Use of Laboratory Animals (publication No. 85-23, revised 1985). Mice were randomly distributed at six per group, an aliquot of 5 × 10^6^ 95-D_NC, 95-D_MELK, H1299_NC, and H1299_shMELK cells was subcutaneously injected into the right flank of each mouse. Tumor volume (mm^3^) was measured with a Vernier caliper and calculated using the formula, (*LW*^2^)/2, where *L* and *W* represented length and width of the tumor.

### RNA-seq data analyses

H1299_NC, H1299_shMELK1, and H1299_shMELK2 (passage 4) were harvested in TRizol for RNA isolation and sequencing. Initial isolates were checked for quality by FastQC software and filtered to remove low-quality calls using default parameters and specifying a minimum length of 50. Processed reads were then aligned to the *Homo sapiens* genome assembly with Cuffmerge software. The levels of mRNA were evaluated by Fragments Per Kilo bases per Million fragments (FPKM) using Cuffquant and Cuffnorm software. The sample correlation analysis was performed using the Pearson coefficient. Cuffdiff software was used to analyze the differential expression, and the default screen standard for differential gene was |log_2_FC | ≥ 1 and *p* value ≤ 0.05. Herein, Log_2_FC referred to Log_2_(fold change). Moreover, log_2_FC ≥ 1 represented up-regulated genes while log_2_FC ≤ −1 represented downregulated genes. The common differential genes from H1299_shMELK1 and H1299_shMELK2 group were collected for subsequent analysis. The gene ontology enrichment analyses were performed using DAVID Bioinformatics Resources 6.8 (https://david.ncifcrf.gov/). The protein–protein interaction was analyzed in STRING website (https://string-db.org/cgi/input.pl) and visualized by Cytoscape software.

### Cell cycle assay

Cell cycle assay was performed by flow cytometry. After treated with OTSSP167 for 24 h, the cells were harvested and fixed with 75% ethanol at 4 °C for 24 h, followed by staining in PI (200 μg/mL RNase, 50 μg/mL PI and 0.1% (v/v) Triton X-100 in PBS) for 30 min. Cell cycle stage was determined by flow cytometry and analyzed by FlowJo software. To identify the role of PLK1 in G2/M phase regulated by MELK, H1299 cells were transfected with siRNA duplexes of PLK1 or NC for 48 h, followed by OTSSP167 treatment for another 24. Finally, the change of cell cycle was checked according to the above procedures.

### Expression level of MMP2, MMP7, and MMP9 in cells by ELISA

MMP2 ELISA kit (Catalog # KHC3081), MMP7 ELISA kit (Catalog # EH328RB) and MMP-9ELISA kit (Catalog # BMS2016-2) were purchased from ThermoFisher SCIENTIFIC company (Shanghai, China). Cell lysis were collected and centrifuged at 12000 rpm for 10 min. After centrifuged, supernatant was collected and ELISA assay was carried out according to the protocols.

### LDH release assay

The level of LDH was measured using Cytotoxicity LDH Assay Kit-WST, which was purchased from Dojindo Molecular Technology Inc. (Tokyo, Japan). H1299 and H1975 cells were treated with or without Emricasan (50 μM) for 24 h, and administrated with OTSSP167 for indicated concentration for another 24 h. The LDH assay was performed according to manufacturer’s instructions.

### Apoptosis assay

The apoptosis rate of cells was analyzed using Annexin V-FITC kits from Beyotime Biotechnology (Hangzhou, China). After treated with OTSSP167 for the indicated time, cells were harvested and washed by PBS three times. The cell pellets were resuspended in an Annexin v-FITC buffer, and incubated on ice for 7 min. Then, PI was added into the mixture. After 3 min, the apoptosis rate of cells was measured by Flow cytometry (FACS Calibur, BD BioSciences). The results were analyzed by FlowJo 7.6 software.

### Statistical analysis

All experiments were repeated three times. The data were expressed as mean ± SD. Statistical analysis was carried out using GraphPad software and comparisons of each group were made by Student’s *t* test using GraphPad software. Results were considered statistically significant at *P* < 0.05.

### Supplementary information

supplemenal materials

## Data Availability

All the data that support the findings of this study are available within the article and its supplementary information files or from the corresponding author upon reasonable request.
