# Correction: MELK is an oncogenic kinase essential for metastasis, mitotic progression, and programmed death in lung carcinoma

**DOI:** 10.1038/s41392-024-01910-4

**Published:** 2024-07-13

**Authors:** Qin Tang, Wan Li, Xiangjin Zheng, Liwen Ren, Jinyi Liu, Sha Li, Jinhua Wang, Guanhua Du

**Affiliations:** 1 The State Key Laboratory of Bioactive Substance and Function of Natural Medicines, Beijing, China; 2 Key Laboratory of Drug Target Research and Drug Screen, Institute of Materia Medica, Chinese Academy of Medical Science and Peking Union Medical College, 100050 Beijing, China

Correction to: *Signal Transduction and Targeted Therapy* 10.1038/s41392-020-00288-3, published online 02 December 2020

After online publication of the article[1] the authors noticed inadvertent errors in the panel of 95-D cells (+ MELK and −siSlug) invasion in Fig. 2J which is partly overlapped with the panel of the 95-D cells (+ MELK and + siSlug) migration in Fig. 2J, and the panel of H1299 cells (siMMP7-1) invasion in Supplementary Fig. S2C which was flipped compared with the panel of 95-D cells (−MELK and −siTwist1) migration in Fig 2J. The panel of the 95-D cells (+ MELK and −siSlug) invasion in Fig. 2J and the panel of H1299 (siMMP7-1) invasion in Fig. S2C were replaced by correct images. The conclusion of the original article or the context of the article was not affected. The authors apologize for any inconvenience caused to the journal and readers. The incorrect part figures and corrected figures and legends are presented as follows.

Incorrect section of Figure 2J:
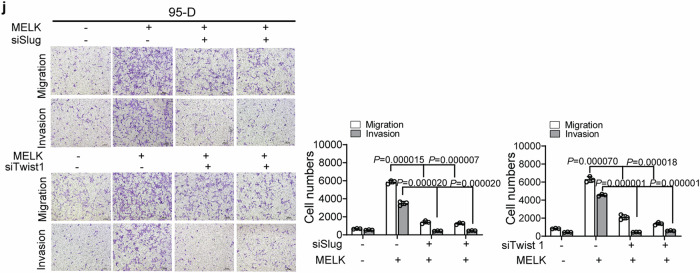


Incorrect section of Supplementary Fig. S2c:
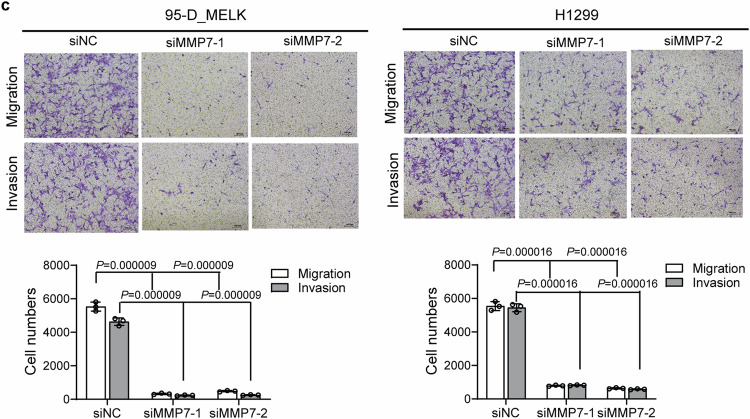



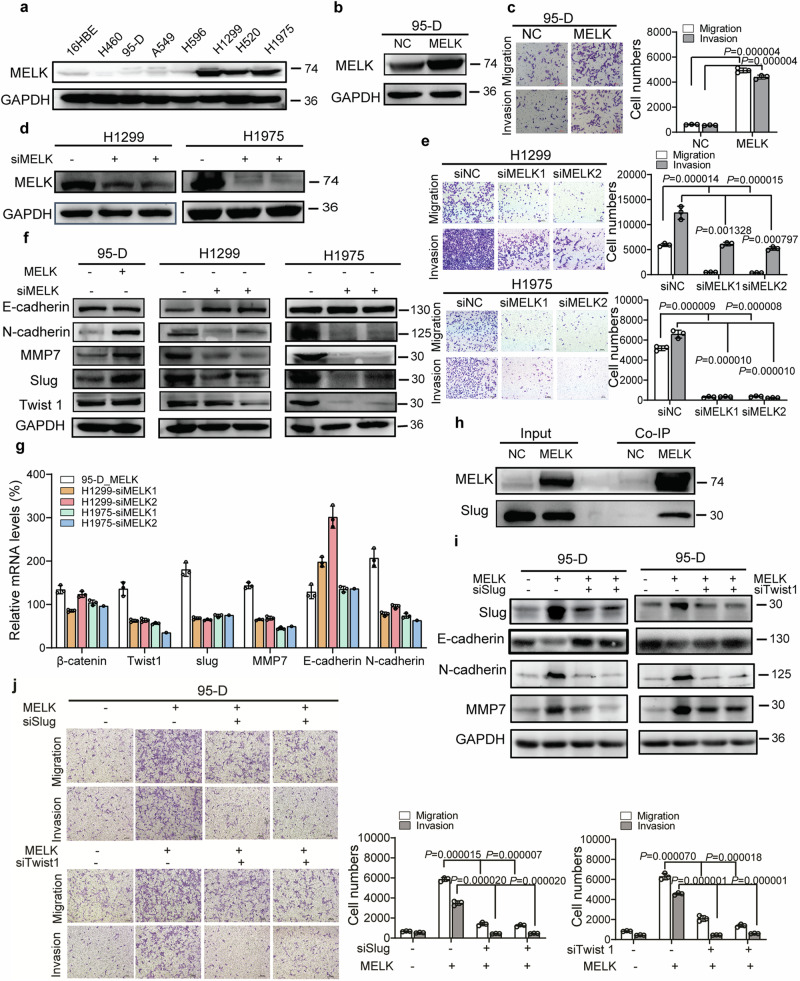


Fig. 2 MELK promoted the migration and invasion of LUAD. **a** MELK expression in normal lung cells and lung cancer cells was analyzed by Western blot. **b** LUAD 95-D cells with relative lower levels of MELK were stably transfected with pCMV6-MELK-Myc-DDK (pMELK) for increasing MELK expression and the results were identified by Western blot. **c** The effects of MELK on migration and invasion in 95-D cells were measured by Transwell experiments. Bars indicates SD, *P* values represented the significant difference between 95-D_MELK and 95-D_NC, Student’s t test. **d** LUAD H1299 and H1975 cells with relative higher levels of MELK were transfected with siRNA duplexes of MELK for 72 h and detected by Western blot. **e** The effects of MELK on migration and invasion in H1299 and H1975 cells were measured by Transwell experiments. After transfected with siRNA duplex of NC or MELK for 48 h, H1299 and H1975 cells were seeded in the Transwell covered with or without Matrigel for invasion or migration assay. Bars indicates SD, *P* values represented the significant difference between siMELK group and siNC group, Student’s t test. **f** The effects of MELK on proteins involved in metastasis were detected by Western blot. H1299 and H1975 cells were transfected with siRNA duplexes of MELK for 72 h and collected for Western blot. **g** The mRNA levels of those protein which were changed by MELK in protein levels. H1299 and H1975 cells were transfected with siRNA duplexes of MELK for 72 h and collected for qRT-PCR. **h** The interaction of MELK and those protein which were changed by MELK in protein levels. 293T cells were transfected with pNC or pMELK for 24 h and collected for Co-IP analysis. **i** The effects of Slug or Twist1 on MELK-inducing pathway of migration and invasion. 95-D_MELK cells were transfected with siRNA duplexes of Slug for 72 h and analyzed by Western blot. **j** Knockdown of Slug or Twist1 reduced the migration and invasion of 95-D with overexpression of MELK. 95-D_MELK cells were transfected with siRNA duplexes of Slug for 48 h, and the transwell experiments were performed. Bars indicates SD, *P* values represented the significant difference between siSlug or siTwist1 group and corresponding siNC group of 95-D_MELK cells, Student’s *t* test
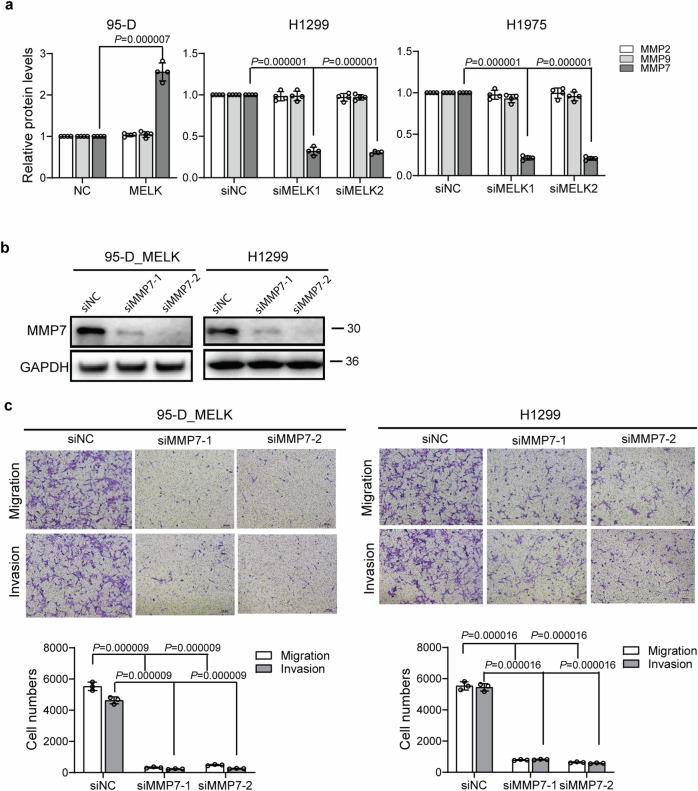


Fig. S2. MELK promoted the migration and invasion of LUAD cells by upregulating MMP7.

**A** The expression levels of MMP7, MMP2 and MMP9 were detected by ELISA assay. ***P < 0.001 represented the significant difference between MELK overexpression or knocking-down group and corresponding NC group. **B** The protein expression of MMP7 was significantly knocked down by MMP7 siRNA. **C** Knocking down of MMP7 expression with siRNA duplexes inhibited the migration and invasion of 95-D MELK and H1299 cells. ***P < 0.001 represented the significant difference between siMMP7 group and corresponding siNC group.

The original article has been corrected.

### Supplementary information

Corrected Fig.S2
