# Supplementary material for: MELK is an oncogenic kinase essential for metastasis, mitotic progression, and programmed death in lung carcinoma
Source: Signal Transduct Target Ther. 2020 Dec 2;5:279. doi: 10.1038/s41392-020-00288-3 (PMC7708490; doi:10.1038/s41392-020-00288-3)
Supplement: Supplementary file 1 — supplemenal materials [file 41392_2020_288_MOESM1_ESM.doc]

Supplementary Materials for

MELK is an oncogenic kinase essential for metastasis, mitotic progression and programmed death in lung carcinoma

Qin Tang^1,2^, Wan Li^1,2^，Xiangjin Zheng^1,2^, Liwen Ren^1,2^, Jinyi Liu^1,2^, Sha Li^1,2^, Jinhua Wang^1, 2, £^, Guanhua Du^1, 2, £^

Correspondence to: [wjh@imm.ac.cn](mailto:wjh@imm.ac.cn); dugh@imm.ac.cn

**This PDF file includes:**

Figures. S1 to S5

Tables S1 to S3


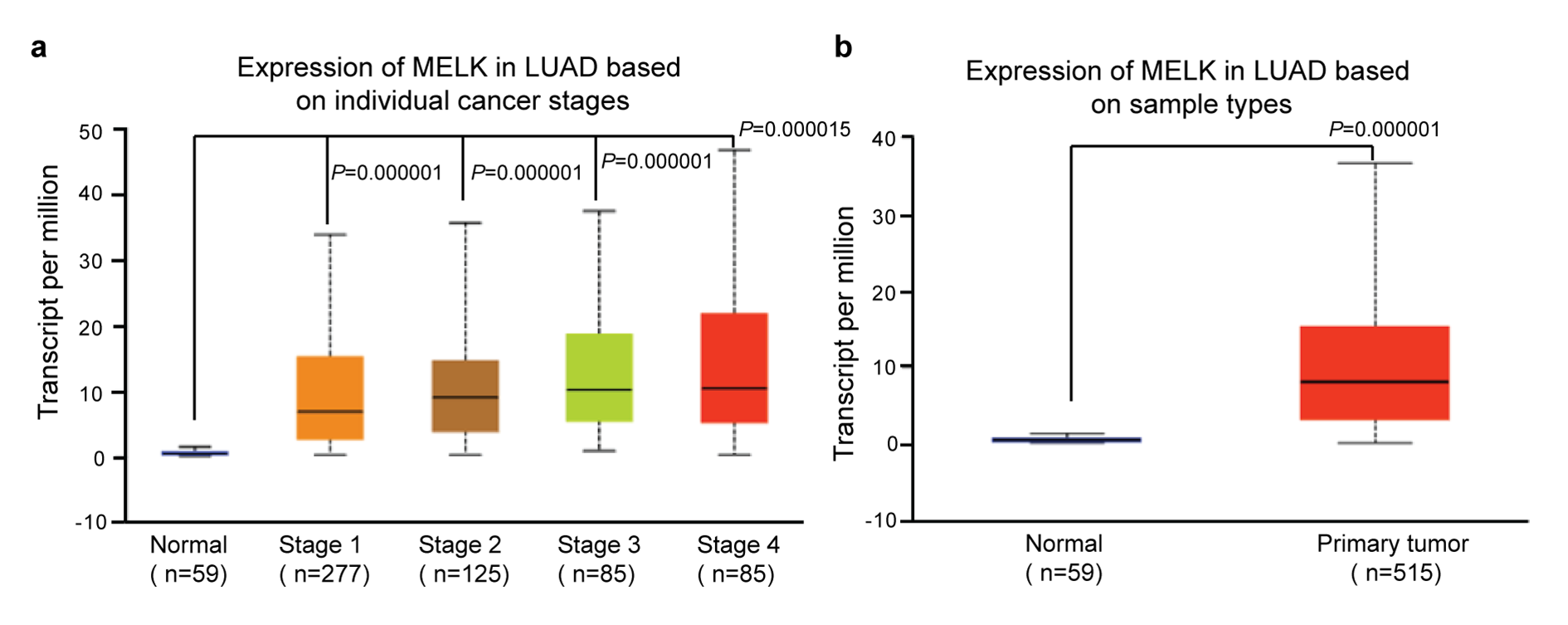


**Fig. S1. The expression of MELK in LUAD based on individual cancer stages and samples types**. **a.** The expression of MELK was increased as development of LUAD. **b.** The expression of MELK in primary LUAD is much higher than that in normal lung tissues. The data were analyzed based on TCGA database. Bars indicates SD, *P* values represented the significant difference between LUAD group and corresponding normal group, Student’s t test.


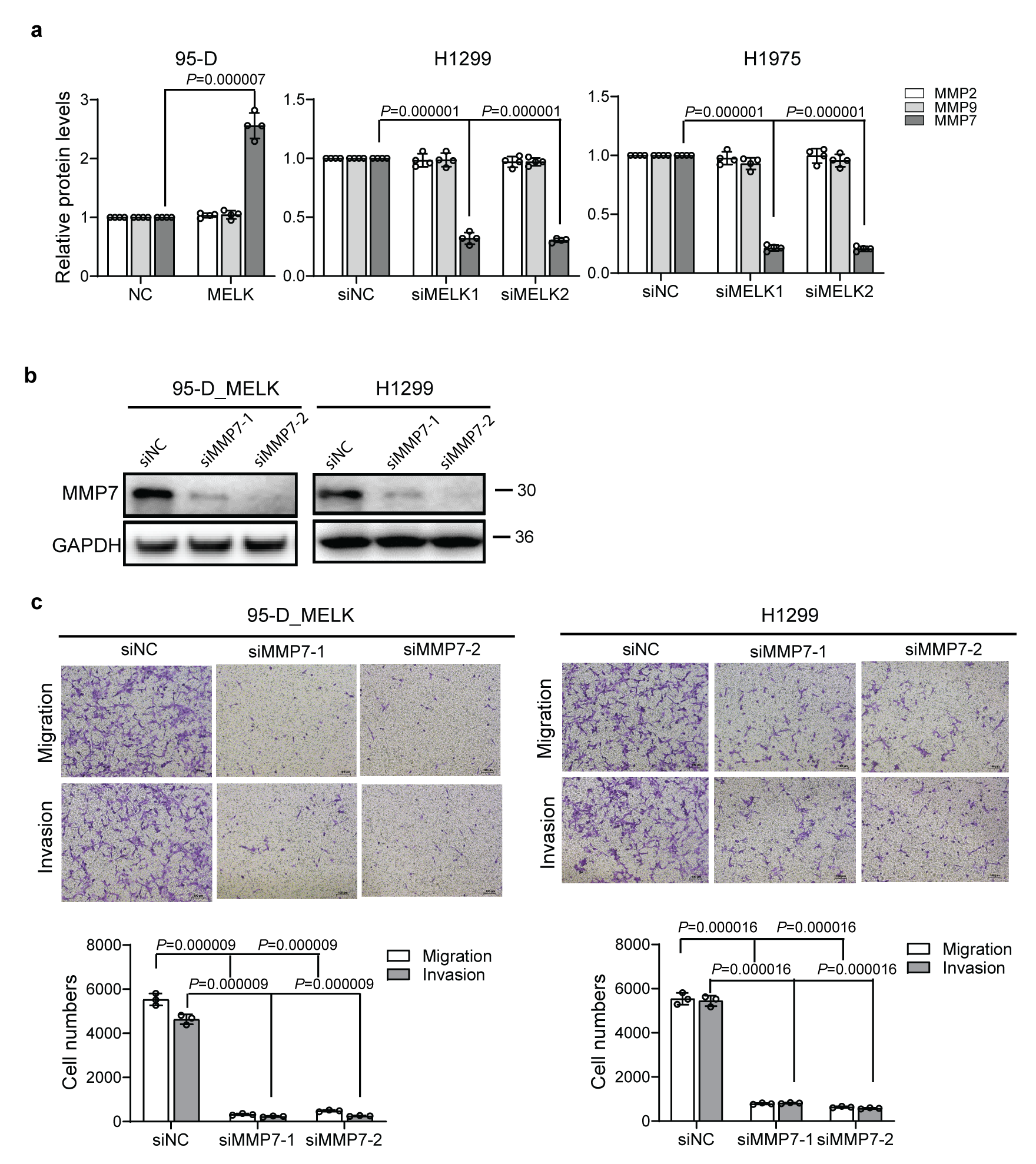


**Fig.S2. MELK promoted the migration and invasion of LUAD cells by upregulating MMP7.**

A. The expression levels of MMP7, MMP2 and MMP9 were detected by ELISA assay. ****P* <0.001 represented the significant difference between MELK overexpression or knocking-down group and corresponding NC group. B. The protein expression of MMP7 was significantly knocked down by MMP7 siRNA. C. Knocking down of MMP7 expression with siRNA duplexes inhibited the migration and invasion of 95-D MELK and H1299 cells. ****P* <0.001 represented the significant difference between siMMP7 group and corresponding siNC group.


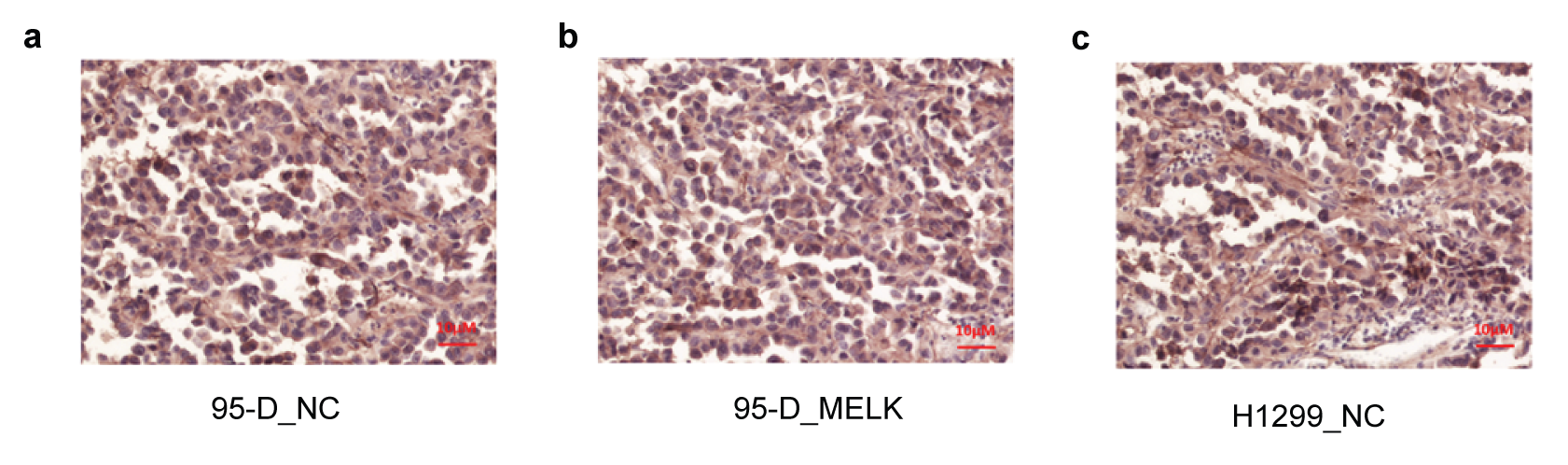


**Fig.S3. Immunohistochemistry analysis of Ki-67 in xenografts**. Immunohistochemistry analysis of Ki-67 in 95-D_NC (**a**), 95-D_MELK (**b**), and H1299 (**c)** xenografts was carried out, which showed no significant difference of Ki67 expression between 95-D_NC and 95-D_MELK cells.


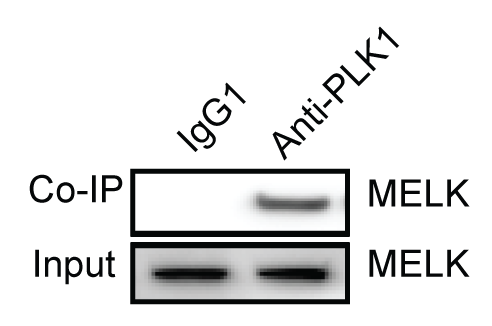


**Fig.S4. Co-IP analysis of PLK1 and MELK**. Cell lysis of 95-D_MELK was collected and immunoprecipitation was performed by normal rabbit IgG and anti-PLK1 antibody, followed by detected by Western blot using anti-MELK antibody. Result showed that MELK interacted with PLK1.


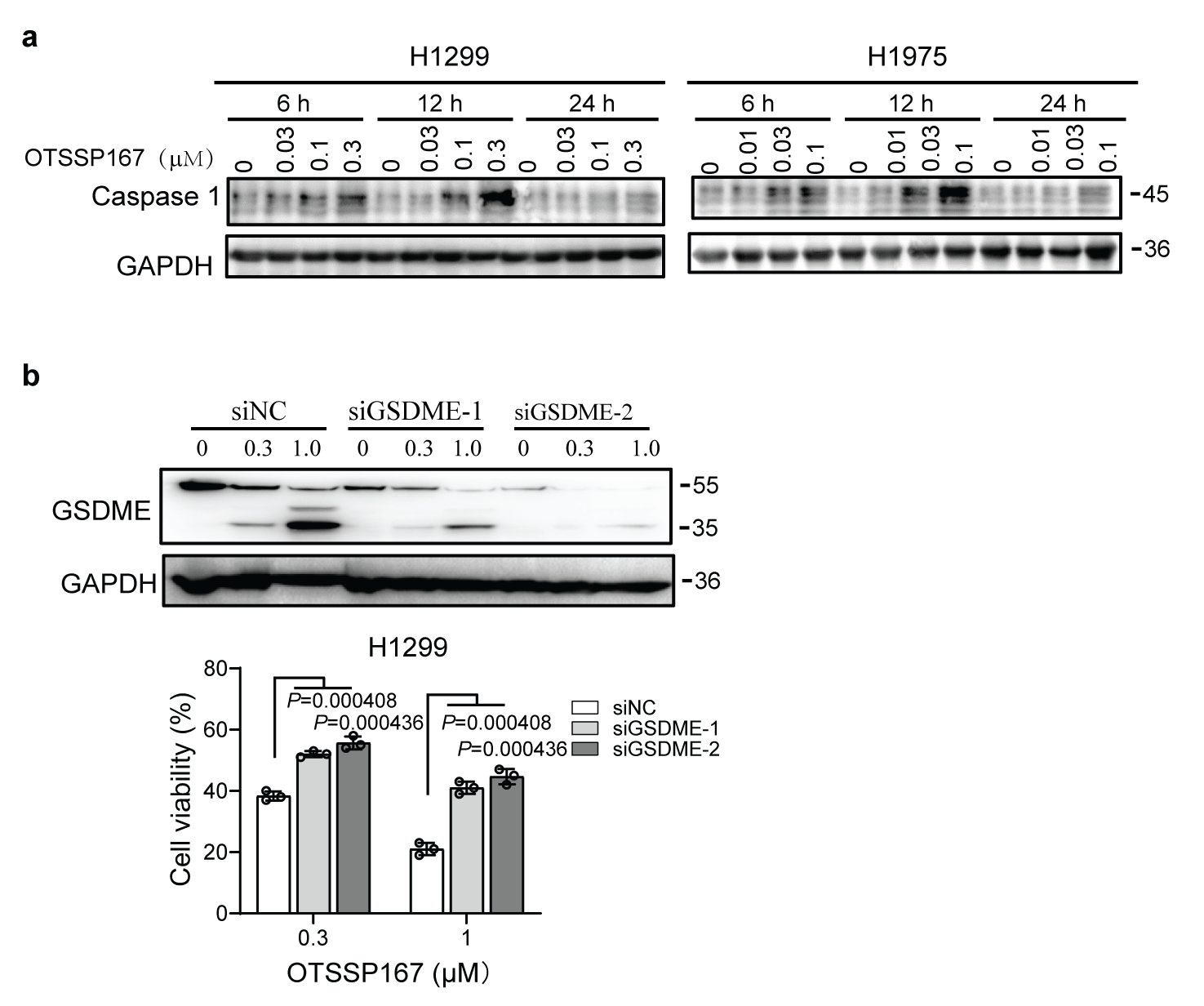


**Fig.S5.** **Inhibition of MELK induced pyroptosis by GSDME**. **a．**The expression of caspase-1 in H1299 and H1975 cells treated with different concentration of OTSSP167 at different time was checked by Western blot. **b**．H1299 cells were pretreated with siRNA duplexes of GSDME for 48 h and treated with OTSSP167 for another 24 h, followed by analysis by MTT assay. Inhibition of MELK induced pyroptosis by GSDME. Bars indicates SD, *P* values represented the significant difference between siGSDME group and corresponding siNC group, Student’s t test.

**Table**

**Table S1** The ID code and dilution of antibodies

| Antibodies | Company | ID code | Dilution |
| --- | --- | --- | --- |
| MELK | Cell Signaling Technology | 2274 | 1:1000 |
| N-cadherin | Cell Signaling Technology | 13116 | 1:500 |
| E-cadherin | Cell Signaling Technology | 3195 | 1:500 |
| Slug | Cell Signaling Technology | 9585 | 1:500 |
| PCNA | Cell Signaling Technology | 13110 | 1:500 |
| SPAG5 | Cell Signaling Technology | 60940 | 1:500 |
| CDC25C | Cell Signaling Technology | 4688 | 1:500 |
| p-T^48^-CDC25C | Cell Signaling Technology | 12028 | 1:500 |
| p-S^216^-CDC25C | Cell Signaling Technology | 9528 | 1:500 |
| PLK1 | Cell Signaling Technology | 4513 | 1:500 |
| p-T^210^-PLK1 | Cell Signaling Technology | 9062 | 1:500 |
| CDK1 | Cell Signaling Technology | 9116 | 1:500 |
| p-T^161^ CDK1 | Cell Signaling Technology | 9114 | 1:500 |
| p-Y^15^ CDK1 | Cell Signaling Technology | 9114 | 1:500 |
| Caspase 3 | Cell Signaling Technology | 9662 | 1:500 |
| Cleaved caspase 3 | Cell Signaling Technology | 9664 | 1:500 |
| Cleaved PARP1 | Cell Signaling Technology | 5625 | 1:1000 |
| GSDMD | Abcame | ab209845 | 1:1000 |
| GSEME | Abcame | ab215191 | 1:1000 |
| GAPDH | Proteintech | 60004-1-Ig | 1:2000 |
| Twist 1 | Proteintech | 25465-1-AP | 1:500 |
| Caspase 3 | Proteintech | 22915-1-AP | 1:500 |
| Ki-67 | Abcame | ab833 | 1:400 |

**Table S2** These siRNA duplexes used in transfection

| Targeted genes | Sence (5' to 3') | Anti-sense (5' to 3') |
| --- | --- | --- |
| Slug-1 | UCUAAUGUGUCCUUGAAGCAA | GCUUCAAGGACACAUUAGAAC |
| Slug-2 | UUCUUUAC AUCAGAAUGGGUU | CCCAUUCUGAUGUAAAGAAUU |
| Twist 1-1 | UAGAGGAAGUCGAUGUACCUU | GGUACAUCGACUUCCUCUAUU |
| Twist 1-2 | UUGAGGGUCUGAAUCUUGCUU | GCAAGAUUCAGACCCUCAAUU |
| PLK1-1 | UUAUCACAGAGCUGAUACCCA | GGUAUCAGCUCUGUGAUAACA |
| PLK1-2 | AUAUUCGACUUUGGUUGCCUU | GGCAACCAAAGUCGAAUAUUU |
| MMP7-1 | GCAUUUCAGGAAAGUUGUAUG | UACAACUUUCCUGAAAUGCAG |
| MMP7-2 | GUAGCAGUCUAGGGAUUAATT | UUAAUCCCUAGACUGCUACTT |
| GSDME-1 | GAUAAGUUACAGCUUCUAAGU | UUAGAAGCUGUAACUUAUCAG |
| GSDME-2 | GCGAUGUACUCAUAGAAGACC | UCUUCUAUGAGUACAUCGCCA |

**Table S3** These primers used in RT-qPCR

| Primers | Sense (5' to 3') | Anti-sense (5' to 3') |
| --- | --- | --- |
| MELK | CACCTCACGGCTACCTAT | TATCACTTGCGGTCACAT |
| Twist 1 | TCCGCAGTCTTACGAGGAGC | GCTTGAGGGTCTGAATCTTGCT |
| Slug | CAGCTCAGGAGCATACAG | GAGGAGGTGTCAGATGGA |
| E-cadherin | CTGAGAACGAGGCTAACG | GTCCACCATCATCATTCAATAT |
| N-cadherin | ATCCTACTGGACGGTTCG | TTGGCTAATGGCACTTGA |
| CCNB1 | AAACTTTGGTCTGGGTCG | GCAATTTGAGAAGGAGGA |
| CCNB2 | ATACCAGTTCCCAAATCC | AGTCAGCTCCATCAAATAC |
| AURKA | CTGTGGCACCCTGGACTA | AGGAGGCTTCCCAACTAA |
| PLK1 | CGAGTTCTTTACTTCTGGCTAT | CTTTATTGAGGACTGTGAGGG |
| CDC25B | ACAGTAGACGGAAAGCACCA | ACAATCACAAACTTATCCACGA |
| SPAG5 | GCAGTGGCAGATGAAGAG | TGTAGCAGGGAACAAAGAC |
| PTTG1 | TCTGTAAAGACCAAGGGAC | ATCATCTGAGGCAGGAAC |
| FOXM1 | CGAAAGATGAGTTCTGATGG | TGTTGATGGCGAATTGTAT |
| TUBB | GCTGGTTGATTCTGTCCTG | TTCTTCTCGGATCTTGCTG |
| GADD45A | AGAGCAGAAGACCGAAAGGA | CAGCAGGCACAACACCAC |
| SKA2 | AACTTTGTATGCCCGCTTTA | CTCCAGGTCTGTTTGCTTCT |
| HIST1H2BH | GCCTGATCCAGCTAAGTCCG | CTCCTTGCGGCTGCGTTT |
| FGF2 | AGGAAGATGGAAGATTACTGGC | CCAACTGGTGTATTTCCTTGAC |
| POM121C | GGCTTCATTACAGTGGTTCAA | CTAACAGTGGGTTGGTGCTT |
| NUP188 | CCTACTGGTCAGGGTATGTC | TCAAGCACGTCAAGAAAGA |
| GAPDH | AAGAAGGTGGTGAAGCAGG | TTGACAAAGTGGTCGTTGAG |
