# Supplementary figures and images for: Correction: MELK is an oncogenic kinase essential for metastasis, mitotic progression, and programmed death in lung carcinoma
Source: Signal Transduct Target Ther. 2024 Jul 13;9:186. doi: 10.1038/s41392-024-01910-4 (PMC11246411; doi:10.1038/s41392-024-01910-4)

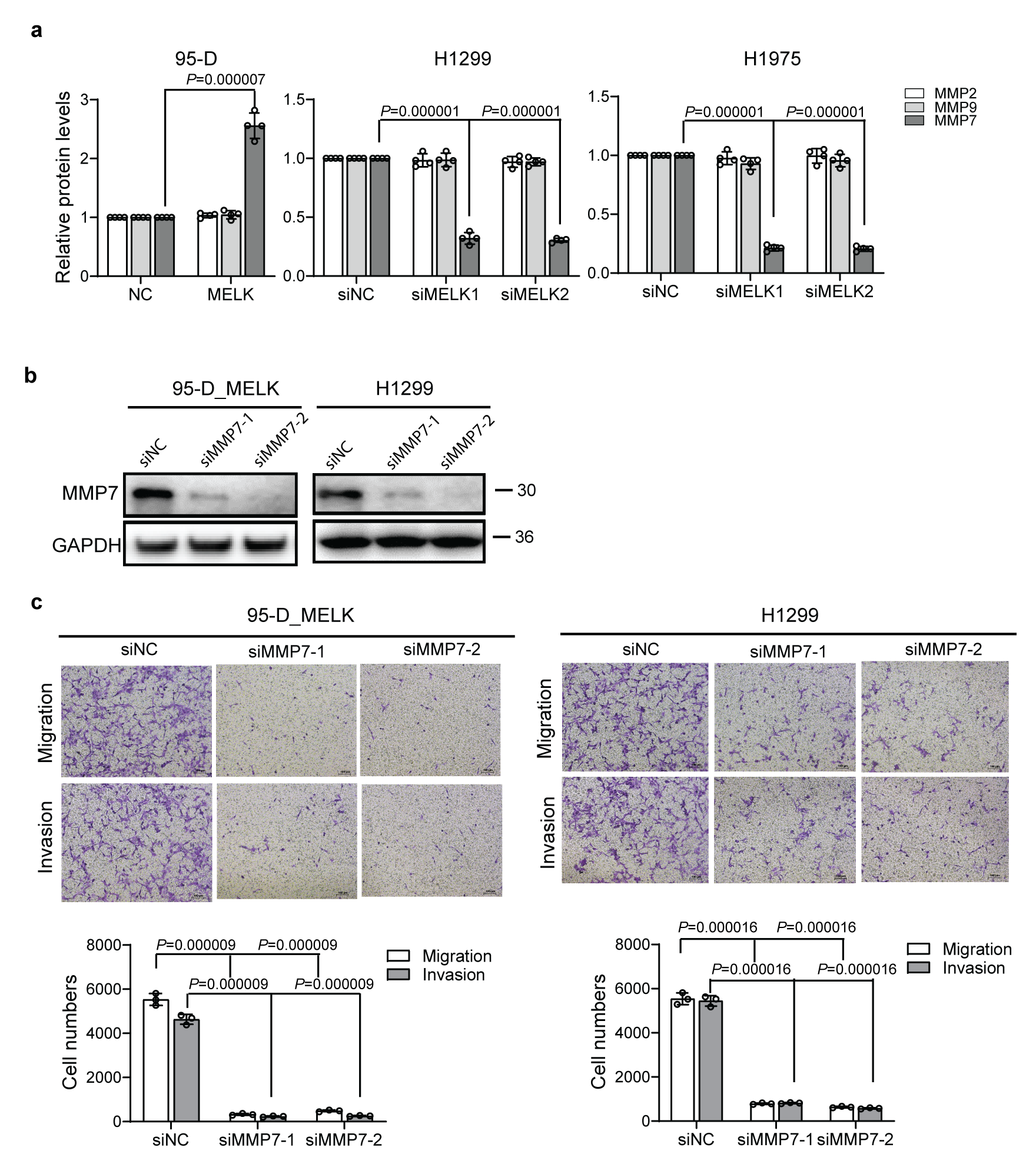

Supplement: Supplementary file 1 — Corrected Fig.S2 [file 41392_2024_1910_MOESM1_ESM.tif]
